# Supplementary material for: Harnessing the Cross-Neutralisation Potential of Existing Antivenoms for Mitigating the Outcomes of Snakebite in Sub-Saharan Africa
Source: Int J Mol Sci. 2024 Apr 11;25(8):4213. doi: 10.3390/ijms25084213 (PMC11050326; doi:10.3390/ijms25084213)
Supplement: Supplementary file 1 [file ijms-25-04213-s001.zip › ijms-2927216-supplementary.pdf]

Table S1A. The median lethal doses of various medically important elapid snake venoms sSA.

Sr. No.	Name of Species	Venom Dose (µg)					Number of survivors					LD50 (µg/mouse) With 95% CI	LD50 (mg/kg) With 95% CI
1	*N. nigricolis*	10	12.5	15.63	19.53	24.41	5	2	1	1	0	9.60<br>7.17-12.84	0.48<br>0.36-0.64
2	*N. palida*	7.70	9.63	12.03	15.04	18.80	5	4	1	0	0	10.76<br>9.94-11.65	0.54<br>0.50–0.58
3	*N. woodi*	7.68	9.60	12	15.00	18.75	5	5	2	1	0	10.90<br>9.39-12.66	0.55<br>0.47-0.63
4	*N. senegalensis*	8	10	12.5	15.63	19.53	5	4	1	0	0	11.18<br>10.33-12.10	0.56<br>0.52-0.61
5	*N. nubiae*	15	18.75	23.44	29.30	36.62	4	2	1	0	0	18.34<br>16.25-20.70	0.92<br>0.81-1.03
6	*N. nivea*	13.2	16.50	20.63	25.78	32.23	5	4	1	0	0	18.45<br>17.04-19.97	0.92<br>0.85-0.99
7	*N. nigricinta*	15	18.75	23.44	29.30	36.62	5	4	1	1	0	21.86<br>19.48-24.54	1.09<br>0.97-1.23
8	*N. katiensis*	18	22.50	28.13	35.16	43.95	5	5	3	1	0	29.62<br>26.83-32.69	1.48<br>1.34-1.63
9	*N. mosambica*	23.44	29.30	36.62	45.78	57.22	5	2	1	0	0	26.62<br>22.67-31.25	1.33<br>1.14-1.56
10	*N. ashei*	23.44	29.30	36.62	45.78	57.22	5	2	2	1	0	26.08<br>19.70-34.55	1.30<br>0.98-1.72
11	*N. annulifera*	32	40	50	62.50	78.13	5	3	1	0	0	42.12<br>38.10-46.55	2.11<br>1.91-2.33
12	*N. anchietae*	27.50	34.38	42.97	53.71	67.14	5	5	4	2	0	51.01<br>16.14-56.38	2.56<br>2.31-2.82
13	*D. polylepis*	4	5	6.25	7.81	9.77	5	5	4	1	0	6.99<br>6.46-7.56	0.35<br>0.32-0.38

The table indicates various dose groups, survival patterns and toxicities of elapid snake venoms from sSA.

Table S1B. The median lethal doses of various medically important viperid snake venoms sSA.

Sr. No.	Name of Species	Venom Dose (µg)					Number of survivors					LD50 (µg/mouse) With 95 % CI	LD50 (mg/kg) With 95 % CI
1	*B. arietans*	8	10	12.50	15.63	19.53	5	5	3	1	0	13.16<br>11.92-14.53	0.66<br>0.60-0.73
2	*B. caudalis*	5.12	6.40	8	10	12.50	4	4	1	0	0	6.86<br>6.10-7.71	0.34<br>0.30-0.39
3	*E. pyramidum*	18.75	23.44	29.29	36.61	45.76	5	2	2	2	1	19.67<br>12.51-30.90	0.98<br>0.63-1.54
4	*E. ocellatus*	10	12.50	15.63	19.53	24.41	5	4	1	0	0	13.98<br>12.91-15.13	0.70<br>0.65-0.76
5	*E. coloratus*	5.15	6.44	8.05	10.06	12.57	4	3	1	1	0	6.86<br>5.92-7.94	0.34<br>0.30-0.40

The table indicates various dose groups, survival patterns and toxicities of viperid venoms from sSA.

Table S2A. Median effective doses and neutralisation potencies of PANAF-Premium antivenom against elapid snake venoms from sSA.

Venom	Challenge dose 3X LD_{50} (µg/mouse)	Amount of antivenom injected in the venom-antivenom mixture (µl)					ED_{50} (µl) With 95 % CI	Potency of antivenom (mg/ml) With 95 % CI	Potency of antivenom (LD_{50}s/ml) With 95 % CI
#N. nigricolis	28.8	111.11	73.96	49.40	32.94	21.97	60.45<br>69.78-52.37	0.318<br>0.275-0.367	33.08<br>28.66-38.19
N. palida	32.28	111.11	73.96	49.40	32.94	21.97	67.37<br>80.77-56.19	0.319<br>0.266-0.383	29.69<br>24.76-35.59
N. woodi	32.7	49.40	32.34	21.97	14.64	9.76	17.93<br>20.71-15.53	1.216<br>1.05-1.40	111.53<br>96.56-128.84
N. senegalensis	33.57	111.11	73.96	49.40	32.94	21.97	60.45<br>69.78-52.37	0.370<br>0.320-0.427	33.08<br>28.66-38.19
N. nubiae	55.02	111.11	73.96	49.40	32.94	21.97	40.34<br>46.59-34.93	0.909<br>0.787-1.05	49.58<br>42.93-57.26
N. nivea	55.35	111.11	73.96	49.40	32.94	21.97	60.45<br>69.78-52.37	0.610<br>0.529-0.705	33.08<br>28.66-38.19
N. nigricinta	65.58	111.11	73.96	49.40	32.94	21.97	91.31<br>151.91-54.89	0.479<br>0.288-0.797	21.90<br>13.17-36.44
N. katiensis	88.86	111.11	73.96	49.40	32.94	21.97	179.22<br>317.15-101.27	0.331<br>0.187-0.585	11.16<br>6.31-19.75
N. mosambica	79.86	166.67	111.11	73.96	49.40	32.94	120.67<br>163.04-89.31	0.441<br>0.327-0.596	16.57<br>12.27-22.39
N. ashei	78.24	166.67	111.11	73.96	49.40	32.94	60.16<br>80.23-45.12	0.867<br>0.650-1.156	33.24<br>24.93-44.33
N. annulifera	126.36	166.67	111.11	73.96	49.40	32.94	**X**	**X**	**X**
N. anchietae	153.03	166.67	111.11	73.96	49.40	32.94	60.16<br>80.22-45.12	1.696<br>1.272-2.261	33.24<br>24.93-44.33
**#D. polylepis*	20.97	111.11	73.96	49.40	32.94	21.97	71.12<br>88.27-57.29	0.197<br>0.158-0.244	28.12<br>22.66-34.91

The above table shows the toxicity neutralisation potencies of PANAF-Premium antivenom manufactured by Premium Serum and Vaccines Pvt. Ltd. The neutralisation potencies were estimated against elapid venoms from different biogeographical with 3X LD_{50} as 'challenge dose' as noted in the table. * Previously published data [1]. Venoms of species marked with '#' were a part of the immunisation mixture. At the same time, the 'X' symbol (and dark red cell colour) represents venom against which the PANAF-Premium failed to offer protection to test animals at 3x challenge dose. Cells marked with dark green colour indicate species against

which significantly higher protection was documented. Cells marked in light green or red colour were respectively found to offer increased or decreased protection.

Table S2B. Median effective doses and neutralisation potencies of PANAF-Premium antivenom against viperid snake venoms from sSA.

Venom	Challenge dose 3X LD_{50} (µg/mouse)	Amount of antivenom injected in the venom-antivenom mixture (µl)					ED_{50} (µl) With 95 % CI	Potency of antivenom (mg/ml) With 95 % CI	Potency of antivenom (LD_{50}s/ml) With 95 % CI
#*B. arietans*	39.48	73.96	49.40	32.94	21.97	14.64	29.99<br>35.88-25.06	0.876<br>0.733-1.05	66.69<br>55.73-79.79
B. caudalis	20.58	166.67	111.11	73.96	49.40	32.94	**X**	**X**	**X**
E. pyramidum	75.12	73.96	49.40	32.94	21.97	14.64	28.80<br>50.90-16.29	1.739<br>0.984-3.07	69.44<br>39.29-122.71
#*E. ocellatus*	41.94	111.11	73.96	49.40	32.94	21.97	40.34<br>54.80-29.69	0.693<br>0.510-0.942	49.58<br>36.50-67.35
E. coloratus	20.58	111.11	73.96	49.40	32.94	21.97	40.34<br>46.58-34.93	0.340<br>0.294-0.393	49.58<br>42.93-57.26

The above table shows the toxicity neutralisation potencies PANAF-Premium antivenom manufactured by Premium Serum and Vaccines Pvt. Ltd. The neutralisation potencies were estimated against viperid venoms from different biogeographical with 3X LD_{50} as 'challenge dose' as noted in the table. Venoms of species marked with '#' were a part of the immunisation mixture. At the same time, the 'X' symbol (and dark red cell colour) represents venom against which the PANAF-Premium failed to offer protection to test animals at 3x challenge dose. Cells marked with dark green colour indicate species against which significantly higher protection was documented. Cells marked in light green or red colour were respectively found to offer increased or decreased protection.

Reference

1. Khalek, I.S., et al., *Synthetic development of a broadly neutralizing antibody against snake venom long-chain alpha-neurotoxins.* Sci Transl Med, 2024. **16**(735): p. eadk1867.
